# Persona-Driven Vignettes: A Novel Needs Assessment and Audit Tool for Faculty Development

**DOI:** 10.7759/cureus.109383

**Published:** 2026-05-21

**Authors:** X. Catherine Tong, Mark Lee, Maria Pratt, Lorand Kristof, Sandra Monteiro, Sean Park, Ruth Chen, Teresa M Chan

**Affiliations:** 1 Department of Family Medicine, McMaster University, Kitchener, CAN; 2 Honours Health Sciences Program, MacPherson Institute, McMaster University, Hamilton, CAN; 3 School of Nursing, McMaster University, Hamilton, CAN; 4 Department of Family Medicine, Toronto Metropolitan University, Brampton, CAN; 5 Department of Family Medicine, McMaster University, Hamilton, CAN; 6 Department of Medicine, McMaster University, Hamilton, CAN; 7 Office of Continuing Professional Development, Faculty of Health Sciences, School of Nursing, McMaster University, Hamilton, CAN; 8 Department of Emergency Medicine, Toronto Metropolitan University, Toronto, CAN; 9 Department of Emergency Medicine, William Osler Health System, Brampton, CAN

**Keywords:** faculty development, health professions education, needs assessment, teacher development, vignettes

## Abstract

Prompted by COVID-19, an academic health sciences center’s health professions faculty development office underwent a digital transformation in 2020. This disruption afforded an opportunity to launch a new approach to comprehensive and rapid needs assessment.

An inter-professional team of health professions educators used a novel storytelling technique to design vignettes featuring fictionalized personas representative of a diverse faculty. Faculty members from a spectrum of teaching practices created vignettes about fictionalized personas based on their lived experiences as faculty. The resulting document was peer reviewed by faculty development team members. Learning objectives were distilled from the vignettes and compiled into a list of items. Redundant items were removed. The items were further analyzed and indexed to reflect the natural life cycle of a faculty member, from recruitment to retirement. Finally, the team reviewed the items and identified resources to address them.

Faculty members created a compilation document that described seven unique faculty journeys through a series of persona-driven vignettes. From this document, three co-authors identified and articulated a collection of learning objectives. After review, 70 learning objectives remained. Most learning objectives (69%, 48/70) could be fully or partially addressed by existing faculty development resources, either locally curated or openly accessible in the literature or online. This process revealed that several gaps in faculty development resources (31%, 22/70) required creation, adaptation, or revision of content.

Using this “vignettes” method, the authors quickly identified gaps in the current resource bank. Like a road map, the list of items defines our tasks to work toward a comprehensive faculty development resource and assess our team’s progress. This method of rapid needs assessment can be replicated in other institutions.

## Introduction

Needs assessment is an essential step in health professional education (HPE) curriculum design [1]. Common faculty development needs of health sciences faculty members are well described in the literature [2]. Yet each institution must assess local needs related to its specific context. With limited resources, an accurate and efficient needs assessment is a perennial challenge in faculty development. Faculty members are asked to complete comprehensive needs assessment surveys [3]. This method can be hampered by the time and skills required for robust survey design, as well as a poor participant response rate. Moreover, the surveys must be re-administered regularly to remain relevant.

In 2020, academic health sciences centers (AHSCs) experienced a major disruption due to the COVID-19 pandemic. Traditional faculty development offerings, including in-person workshops and longitudinal programming, were no longer possible. The office of Continuing Professional Development (CPD) in the Faculty of Health Sciences (FHS) at McMaster University endeavored to undergo a rapid transformation to digital offerings [3,4]. In that process, it became clear that, in addition to new learning needs related to virtual teaching, it was also necessary to continue to address perennial faculty learning needs that remain essential in their work during the pandemic and post-pandemic. Moreover, the CPD office must deliver programming through a just-in-time digital delivery [5]. For example, newly recruited faculty members still needed to learn how to facilitate small groups, whether virtually or in person. Ideally, all resources should be presented together in an easily accessible format. As such, the CPD office must complete a rapid inventory on pre-existing resources, keep what’s current, and aim to curate a comprehensive collection online.

The task to contextualize faculty development into the local academic setting was urgent, important, and challenging. We are a small team of faculty members and staff working with the CPD office with a modest budget. The office oversees the faculty development programming across all of McMaster’s HPE programs, including medicine, nursing, rehabilitation sciences, midwifery, physician assistant, and a multitude of Bachelor of Science programs. In addition, the faculty members work in four distributive campuses and several clinical teaching sites throughout Central and West Ontario. In total, the program supports over 3,000 faculty members with highly heterogeneous academic and clinical roles in diverse contexts. In the rapidly changing landscape of health professions education during the pandemic, it would be challenging to conduct a rapid large scale needs assessment project using conventional methods. We developed a new strategy to investigate faculty needs in this study.

Design thinking is a methodology for defining a challenge to address, surfacing the needs of people (users) through ethnographic field work, and designing and iteratively testing interventions to address the identified challenge and user needs [6]. In design thinking, “vignettes” and short stories are well-described methods in needs assessment [7]. Ethnography is traditionally used in design processes as researchers are often outside the culture of those being designed for or with. However, autoethnography includes the researcher or designer in the process as it allows for “epiphanies that stem from, or are made possible by, being part of a culture or by possessing a particular cultural identity” [8]. Narrative and vignettes are useful tools in conducting autoethnography, as they provide “aesthetic and evocative thick descriptions of personal and interpersonal experience” [8]. In this study, we use autoethnography to create vignettes that are snapshots reflecting an individual's experience or situation [9]. We then leverage design thinking to plan CPD programs.

## Materials and methods

In 2020-2021, we recruited six health sciences faculty members in medicine, nursing, rehabilitation sciences, and research, both clinical and non-clinical, representing a spectrum of teaching practices to create vignettes. The faculty members were recruited based on their roles and profiles that were representative of common faculty portfolios. They were invited to write vignettes, i.e. fictionalized personas, based on their own lived experiences as faculty members in the third person. Faculty members were prompted to write about their experiences from onboarding to the present, with a fictional name assigned to the story, and to use it as a vehicle to narrate pertinent topics and moments in their career development as they recall them. They were also prompted to include significant experiences that were common among their peers, if relevant. During this writing process, faculty members identified professional development needs retrospectively. They described common challenges in managing many professional roles at various stages. In addition, the vignettes also captured learning needs in navigating the tenure and promotion process. When the study team members peer-reviewed the vignettes, they further complemented learning needs by generating additional items related to teaching logistics, support, and resources.

We followed a five-step process to identify the unique and common needs of our diverse faculty personas and then used this list to audit our existing faculty development resources. To illustrate the process with an example, “Lindsay” is a part-time clinical faculty member. Her journey starts with her recruitment into teaching faculty as a new graduate, through her growth as an academic leader, and ends with her contemplating retirement. Throughout her career, Lindsay encountered many questions, such as: “how to give feedback to learners,” “time management for the busy clinician teacher,” and “what are the key competencies for a program director/academic leader.” The team took advantage of many curated resources and mapped them to each individual question asked by Lindsay. For any given question, the answers could be in the form of a website, a podcast, an article, or a YouTube video. Lindsay shared some common learning needs with many other vignettes while retaining several unique needs in her path.

## Results

**Figure 1:**
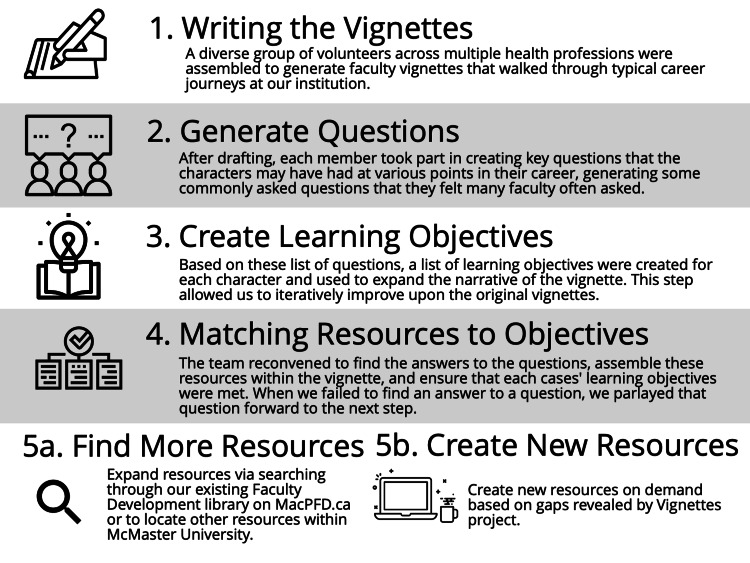
A five-step process was used to identify the unique and common needs of faculty personas and to audit existing faculty development resources.

The five-step process is outlined in Figure 1. In step 1, six faculty members created a compilation document of 5,370 words that described seven unique faculty journeys. On average, each faculty journey consisted of an average of 767 words (SD = 643.34) and contained descriptions of four key phases of each persona’s career, including “onboarding,” “early career,” “mid-career,” and “late career.” There are many commonalities across the personas in their experiences, as well as a diversity of challenges they encountered in each of their contexts.

In step 2, based on this master document of all the vignettes, following design thinking’s human-centered approach, all members collaborated to generate natural questions that may occur at each level for each persona based on the vignette they experienced.

**Figure 2:**
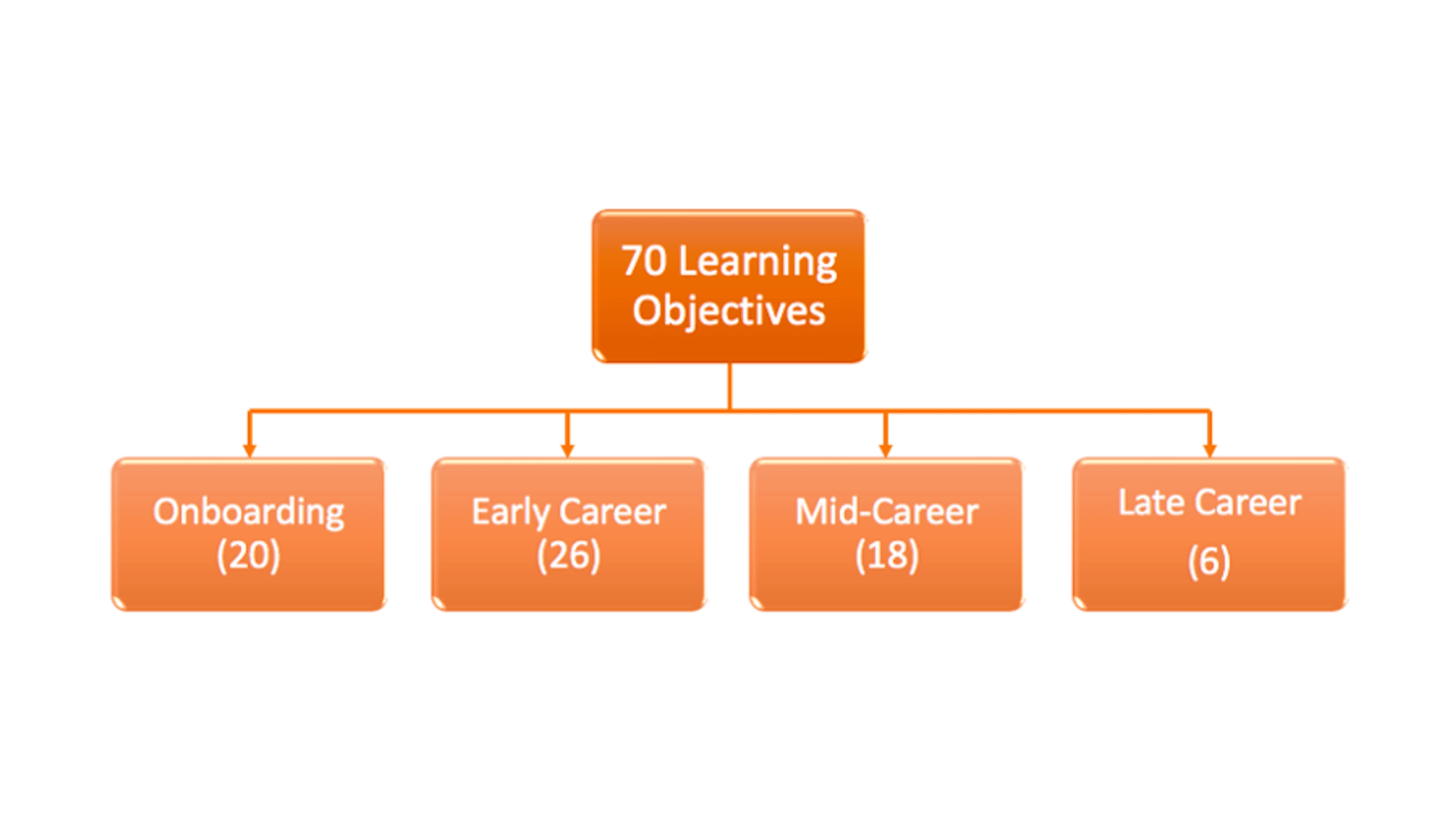
A summary of learning objectives.

In step 3, three members of our team (XCT, ML, and TMC) further reviewed and outlined a collection of learning objectives that were linked to each career stage and the questions that naturally emerged for the persona characters within their vignettes. These were then coalesced and combined to ensure that there were no redundancies. After review, 70 learning objectives remained, with each item indexed into “onboarding,” “early career,” “mid-career,” and “legacy planning/retirement” (Figure 2).

In step 4, the team worked to match existing resources to the objectives. An example of these resources would be answers to questions like “How do you become a community preceptor for McMaster University” (onboarding) and “what can I do with my teaching feedback” (early career). Many items could be fully or partially addressed by existing faculty development resources, either already curated locally or found readily in online open-access resources. However, significant addition, adaptation, and revision of content were needed to fully address 22 of the 70 items.

**Figure 3:**
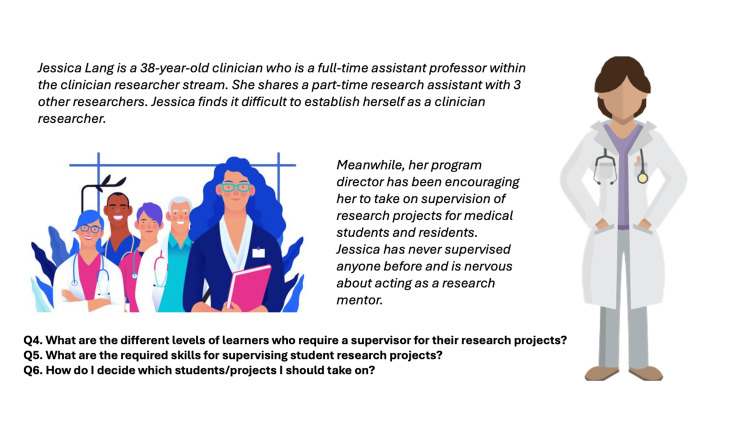
The vignette of "Jessica," a full-time clinical research faculty member, illustrates common questions related to research supervision.

In step 5, the team sourced or created new materials to address these items, an example of which is shown in Figure 3. In this example, a full-time research faculty member wonders about various levels of learners who require research supervision. As a result of identifying this gap through this project, a live workshop on this topic took place on November 16, 2020. Shortly after, a recording from the discussion was embedded into a CPD resources web page.

## Discussion

In this way, we have leveraged the vignettes document to a web page for the faculty to use as a tool for self-directed needs assessment. This tool helps faculty members to perceive previously unperceived needs. We called it the “Journey” website (journey.macpfd.ca). The website lists all seven vignettes and takes the user through each of their unique faculty journeys. Resources curated by the CPD team, as well as those mapped from free open-access sources on the web, are mapped to each question.

Since the initial launch of the Journey website, the team has presented the website to several faculty leads to solicit feedback. For this part of the project, we received an exemption from a full research ethics review from the Hamilton Integrated Research Ethics Board. We have collected a variety of comments from faculty leads who are familiar with the challenges of recruiting, onboarding, engaging, and mentoring faculty members throughout their careers. We engaged with senior academic leads, including department chairs, educational scientists, and representatives from the school of medicine, nursing, and rehabilitation sciences, to review our product. The participants reported that the product was “very interesting”; “would have been useful as (they were) progressing”; “a fabulous resource to share”; “engaging”; “much better than (a previous resource)”; “useful to junior faculty”; and “impressed by the amount of resources captured.” At the same time, the participants identified areas for improvement to make it more applicable, including “add more resources to later-career stage”; “there could be more synthesis (in addition to) a list of URLs”; and “(important to) orient chairs/division directors to integrate this.” To incorporate the feedback into our first version of this product, we have planned the following improvement steps: specifically invite later-career faculty members to contribute to the legacy-planning/retirement group of learning objectives; add high-level summaries to synthesize the links listed to each question asked in the vignettes; and link this website to high-traffic resources pages.

Although the project was completed at McMaster University, several team members later became involved with a new medical school in Ontario in 2024. The Toronto Metropolitan University (TMU) School of Medicine (SOM) launched its undergraduate and postgraduate programs in 2025. This presented a new challenge in faculty recruitment and development, as the staff and physicians working in these centers may have had limited prior involvement in teaching. In this case, the faculty development team again needed to assess and support faculty learning needs. Additionally, innovative curricula at the new medical school necessitate the creation of new resources and underline the importance of implementing new methods of faculty development. Using the approach of persona-driven vignettes specifically aimed at TMU SOM faculty provides an efficient and novel way to develop local faculty. We reviewed and updated our resources on the current Journey website and added a scenario where our character joins the faculty of a new medical school (see Part Time Clinical Faculty: https://journey.macpfd.ca/lindsays-journey).

In our study, we found that overall, our participants had similar faculty development needs with what are commonly described in literature. Our project successfully connected locally relevant resources to support faculty needs whenever possible. We acknowledge that our study is limited by the small sample size of faculty members who contributed to the vignettes. While the vignettes methodology is applicable to various institutional contexts, the personas, the specific needs, and the linked local resources would vary based on institutional context.

## Conclusions

The persona-driven vignettes method for needs assessment was appropriate for the faculty development team at our large interprofessional academic health sciences institution, given the urgency imposed by the pandemic and the limited resources available to the team. Through autoethnography and asynchronous web-based presentation, the final product remains authentic and easily accessible. Upon building the first version, the product is amenable to ongoing review and continuous iteration to remain contemporary and effective. As AHSCs continue to grapple with the long shadow cast by the pandemic, this method can be adopted at any institution in need of rapidly conducting a needs assessment and updating its resources for faculty members with dynamic learning needs.
